# Estimating age-mixing patterns relevant for the transmission of airborne infections

**DOI:** 10.1016/j.epidem.2019.03.005

**Published:** 2019-09

**Authors:** Nicky McCreesh, Carl Morrow, Keren Middelkoop, Robin Wood, Richard G. White

**Affiliations:** a Department of Infectious Disease Epidemiology, London School of Hygiene and Tropical Medicine, London, UK; b The Desmond Tutu HIV Centre, Institute of Infectious Disease and Molecular Medicine and Department of Medicine, University of Cape Town, Cape Town, South Africa

**Keywords:** Social contact, Airborne infection, Mathematical modelling, Tuberculosis, Age-mixing

## Abstract

**Introduction:**

Age-mixing patterns can have substantial effects on infectious disease dynamics and intervention effects. Data on close contacts (people spoken to and/or touched) are often used to estimate age-mixing. These are not the only relevant contacts for airborne infections such as tuberculosis, where transmission can occur between anybody ‘sharing air’ indoors. Directly collecting data on age-mixing patterns between casual contacts (shared indoor space, but not ‘close’) is difficult however. We demonstrate a method for indirectly estimating age-mixing patterns between casual indoor contacts from social contact data.

**Methods:**

We estimated age-mixing patterns between close, casual, and all contacts using data from a social contact survey in South Africa. The age distribution of casual contacts in different types of location was estimated from the reported time spent in the location type by respondents in each age group.

**Results:**

Patterns of age-mixing calculated from contact numbers were similar between close and all contacts, however patterns of age-mixing calculated from contact time were more age-assortative in all contacts than in close contacts. There was also more variation by age group in total numbers of casual and all contacts, than in total numbers of close contacts. Estimates were robust to sensitivity analyses.

**Conclusions:**

Patterns of age-mixing can be estimated for all contacts using data that can be easily collected as part of social contact surveys or time-use surveys, and may differ from patterns between close contacts.

## Highlights

• Airborne infection transmission can occur between anybody sharing indoor space.
• We demonstrate a method for calculating age-mixing patterns for these contacts.
• It only requires data that can be easily collected during social contact surveys.
• Age-mixing patterns for these contacts may vary from those typically used in models.

## 1 Introduction

Differential patterns of contact between different age groups in a population can have a range of important effects on infection and disease dynamics. Arregui et al. (2018) demonstrated that simulating patterns of age-mixing alters patterns of *Mycobacterium tuberculosis* (*Mtb*) transmission between age groups, and generates differences in age-specific incidence rates of infection and tuberculosis disease (Arregui et al., 2018). This has implications for the age targeting of vaccines or other intervention measures. In another mathematical modelling study, Winter et al. (2018) showed that incorporating age-mixing patterns decreased the projected estimates of reductions in congenital rubella syndrome attained from introducing public-sector rubella vaccination (Winter et al., 2018).

Most modelling studies obtain estimates of age-mixing patterns from empirical studies of ‘close’ contacts – face-to-face contacts involving an exchange of words and/or touch (e.g. (Worby et al., 2015)). This reflects the data that have been collected in empirical social contact studies (Van Hoang et al., 2019). In addition, age-mixing patterns that may approximate those of close contacts have also been estimated using time-use data (Zagheni et al., 2008). It is plausible that these close contacts approximate reasonably well the contacts that are most relevant for the transmission of direct contact and droplet infections such as influenza (Brankston et al., 2007) (although they will still miss potentially effective contact occurring between strangers in crowded locations such as closely-packed public transport). For airborne infections such as *Mtb* however, transmission does not require such close contact, and transmission can occur between anyone ‘sharing air’ in insufficiently ventilated indoor spaces, regardless of whether or not conversation or physical contact occurs (Raffalli et al., 1996). In line with other studies, we define indoor contacts who do not meet the criteria for close contact as ‘casual contacts’ (Dodd et al., 2015; McCreesh and White, 2018). Due to higher contact numbers and saturation of close contacts, these casual contacts may be responsible for the majority of infection in high incidence settings (McCreesh and White, 2018).

While some studies have collected data on casual contact numbers (Dodd et al., 2015; McCreesh et al., 2016; Johnstone-Robertson et al., 2011; Mikolajczyk and Kretzschmar, 2008), and one study has estimated the casual contact age-mixing patterns in certain key location types (Andrews et al., 2014), to our knowledge no study has attempted to estimate overall patterns of age-mixing among casual or all (close and casual) contacts. Studies which included questions on casual contact numbers have not asked about the age of casual contacts, and it is unlikely that respondents would be able to estimate and recall the ages of people that they did not speak to. This means that age-mixing patterns among casual contacts cannot be estimated using direct methods.

In this paper, we demonstrate an indirect method of estimating age-mixing patterns among casual contacts, using data that can be easily collected as part of social contact or time-use surveys. This method requires very little additional data collection, and can generate more realistic patterns of age-mixing for use in the parameterisation of mathematical models of tuberculosis and other airborne infections.

## 2 Methods

### 2.1 Study community

The social contact study was conducted in a peri-urban township near Cape Town, South Africa in 2010 (Johnstone-Robertson et al., 2011). A census of the study community in 2008 estimated the resident population to be 14540, with 18% aged <15 years, 62% aged 15–34, and 20% age 35 + . 54% of the population were male.

### 2.2 Data collection

Participants were randomly selected, stratified by age group (0–5, 6–11, 12–17, 18–23, 24–29, 30–40, and >41 years) from a census of the study community conducted in 2008. Participants were recruited at home, and houses were visited a maximum of four times to recruit selected individuals. Residents who had emigrated since the census were replaced, with the replacement selected at random from the same age strata. Residents who could not be found or did not consent were not replaced. Residents under the age of three had not been alive at the time of the census. Participants aged 0–5 were therefore selected from the children of randomly selected women aged 15–45 years.

Respondents were asked to complete a diary over a 24 h period (5am-5am), listing all locations where they had close social contact with another person (close contacts are defined as contacts with face-to-face two-way conversation involving three or more words and/or contact involving physical touch). For each location, respondents were asked whether the location was inside or outside the study community, whether the location was indoors or outdoors, the location type (e.g. ‘your household’, ‘train’, ‘church’), the time spent at the location, the time of day the location was visited, details of close contacts met there (age, gender, touch y/n, and first time today y/n), and the number of people present at the location who did not meet the criteria for close contact (referred to here as ‘casual contacts’). Figure S1 shows the social contact diary.

The study was approved by the Human Research Ethics Committee of the University of Cape Town. Written informed consent was obtained from all participants. Parental/guardian consent was obtained for participants under 18 years of age, and signed assent forms were obtained from adolescents aged 12–17 years.

The social contact study methods are described in full in previous publications (Johnstone-Robertson et al., 2011; Wood et al., 2012).

### 2.3 Analysis

Contacts occurring outdoors were excluded, as outdoor *Mtb* transmission is believed to be greatly limited by ventilation and ultraviolet light (Segal-Maurer and Kalkut, 1994). Incomplete diaries were also excluded, as were contacts where the location type was missing. All estimates were weighted by age, sex, and day of the week. As durations of building visits were recorded categorically, category mid-points were used in calculations (e.g. a duration of 7.5 min was used for reported visits of 0–15 min). A duration of 18 h was used for visits that were reported to have lasted more than 12 h. Locations where a respondent reported that more than 50 casual contacts were present were recoded as 50 casual contacts present.

Estimates of mean close contact numbers, mean close contact time, and close contact rates by age were calculated directly from data on people spoken to or touched. Repeated contacts were only counted once.

Data on the estimated ages of casual contacts were not collected, and therefore indirect methods were used to estimate mean casual contact numbers, mean casual contact time, and casual contact rates between different age groups. The method used was as follows:

1) The average amount of time spent by respondents in each age group, in each type of location, was calculated. From this, the average amount of time spent by members of the study community in each age group, in each location, was calculated, taking into account the sampling fraction in each age group. This was this used to estimate the age distribution of people who were present at each location type.
2) For each respondent and individual location visited, the number of casual contacts that the respondent reported were present at that location was combined with the estimated age distribution of people present at that location type, to give the numbers of casual contacts in each age group present at that location.
3) For each respondent and individual location visited, the contact time by age group at that location was estimated by multiplying the duration of time the respondent reported spending at the location with the estimated number of casual contacts in each age group present at that location
4) For each respondent, estimated casual contact numbers and times by age group were summed over all locations that the respondent reported visiting
5) Results were averaged by age group, and mean rates calculated

In this paper, the phrase ‘all contact(s)’ is used to denote number of contacts or contact time of close and casual contacts combined. ‘Total contact(s)’ is the number of contacts or contact time with people in all age groups combined.

Contact numbers and contact times for all contacts were calculated by summing the results for close and casual contacts. All visits to houses (‘your household’, ‘other household on your plot’, and ‘other household off your plot’) were combined into a single location type).

The method used for calculating age-mixing among casual contacts assumes that there is no difference between individual locations of each location type in the age distribution of people who visit the location. For instance, it assumes that churches are not divided into some churches that are visited predominantly by younger people, and some churches that are visited predominantly by older people. For schools in particular, we know that this assumption is inaccurate. In South Africa, schools are divided into primary schools for children aged 7–13 years, and secondary schools for children aged 14–18 years. Casual contacts in schools will therefore be more likely to be within age groups, than between them. Furthermore, much of the indoor contact in schools will occur in classrooms, which are separated by age in one-year bands. For this reason, we also estimated adjusted casual contact patterns, where we assumed that patterns of age-mixing between casual contacts in schools were the same as patterns of age-mixing between close contacts in schools.

Mean contact rates and mean contact time between each individual in the study community by age group were calculated for all estimates, by dividing contact numbers and contact time by the study community population size in the contact age group.

Symmetrical contact rates between people in age group *i* and people in age group *j* were calculated by averaging estimated rates between respondents in age group *i* and contacts in age group *j*, and estimated rates between respondents in age group *j* and contacts in age group *i*.

### 2.4 Sensitivity analyses

Three sensitivity analyses were conducted. In the first, location types that fewer than 20 people reported visiting were excluded from the analysis, as estimates of the age distribution of casual contacts in these locations may be inaccurate. Comparing estimated casual age-mixing patterns calculated with and without excluding these locations gives an indication of the potential magnitude of any inaccuracies in the age-mixing estimates.

Secondly, the proportions of people present in each type of location who were in each age group were estimated *by time of day* (morning, afternoon, or evening). Where location visits were reported to have occurred during two or three time periods, it was assumed that half or a third respectively of the time spent in the location occurred during each time period. Comparing the age-mixing patterns estimated in this way with the main estimates of age-mixing patterns gives an indication of whether or not the estimated are biased by any tendency for people in different age groups to visit locations at different times of day. As the numbers of people reporting visits to each location type and time period is lower than the numbers reporting visits to each location type at any time of day, this approach is not used for the main analysis.

In the third sensitivity analysis, the age distribution of casual contacts in workplaces was estimated from the age distribution of close contacts that respondents reported contacting in workplaces. This method has previously been used to estimate age-mixing patterns in five key location types in the study community (Andrews et al., 2014). This sensitivity analysis was conducted because for some workplaces, not all individuals who visit the location would class it as ‘your work’. For instance, shops will be classed as workplaces by the shop employees, but as shops by customers.

## 3 Results

### 3.1 Total contact numbers

Diaries from six participants were excluded as they were not completed. More than 50 casual contacts in the same location were reported by 37 respondents in 48 locations. These locations were recoded as having 50 casual contacts. Data on the location type was missing for 21 contacts occurring in indoor locations. These observations were excluded from the analysis. This left data on 8807 close contacts in 2414 indoor locations from 565 respondents. A full description of respondent characteristics is given in Johnstone-Robertson et al (2011) (Johnstone-Robertson et al., 2011).

Table 1 shows the number of respondents, mean indoor close and casual contact numbers per respondent per day, and mean total close and casual contact time per respondent per day, by respondent age group.

**Table 1:** Mean numbers of indoor close and casual contacts per day and mean contact time with close and casual contacts per day, and the proportions that occurred within the study community, by respondent age group.

Respondent age (years)	Number of respondents	Mean number of indoor contacts per respondent per day (proportion occurring within study community)		Mean minutes of indoor close contact time per respondent per day (proportion occurring within study community)	
		Close contacts	Casual contacts	Close contacts	Casual contacts
**0-4**	66	9 (0.87)	13 (0.63)	72 (0.95)	38 (0.85)
**5-9**	63	10 (0.9)	18 (0.7)	70 (0.95)	62 (0.77)
**10-14**	65	12 (0.84)	26 (0.6)	86 (0.9)	76 (0.61)
**15-19**	74	14 (0.87)	31 (0.58)	96 (0.96)	83 (0.72)
**20-24**	81	11 (0.7)	31 (0.33)	64 (0.78)	68 (0.36)
**25-29**	59	12 (0.62)	31 (0.24)	71 (0.71)	73 (0.2)
**30-34**	48	11 (0.61)	30 (0.34)	66 (0.65)	76 (0.28)
**35-39**	25	9 (0.86)	14 (0.44)	71 (0.89)	40 (0.33)
**40-44**	45	10 (0.71)	26 (0.34)	63 (0.82)	40 (0.43)
**45+**	39	8 (0.85)	13 (0.43)	56 (0.93)	37 (0.61)
**Total**	565	11 (0.74)	26 (0.39)	71 (0.82)	64 (0.42)

**Fig. 1:**
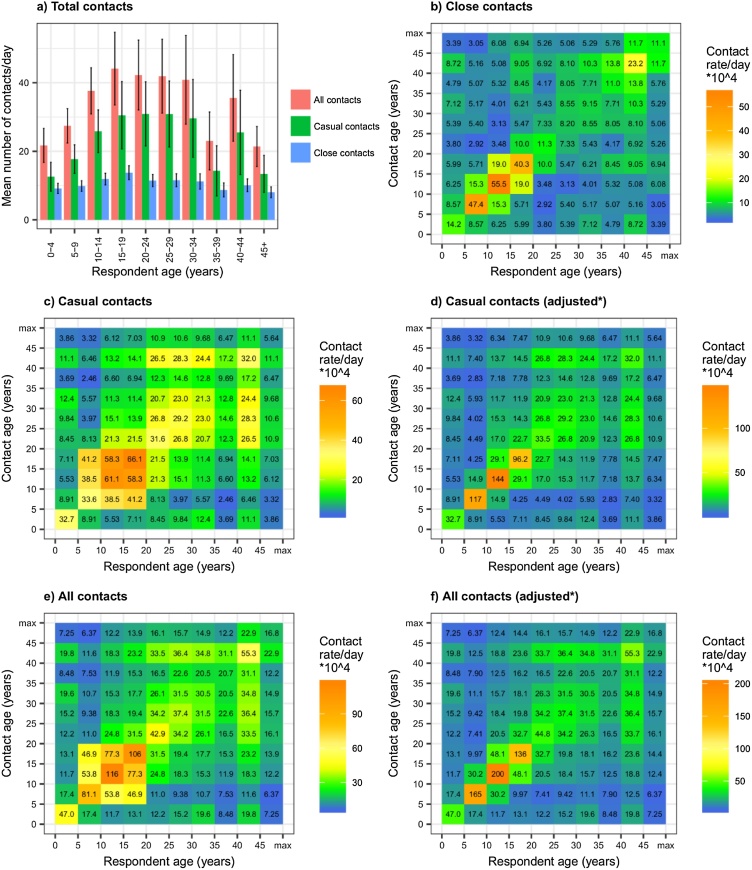
**Total contact numbers per day by respondent age group, and daily contact rates between close, casual and all contacts by age group.** Rates are the estimated rate of contact per day between each individual in age group *a* with each individual in age group *b* (assuming a closed community). Graph a) shows total contact numbers by respondent age. Error bars show 95% confidence intervals, graph b) shows close contact rates, graphs c) and d) show casual contact rates, and graphs e) and f) show rates in all contacts. *Age mixing patterns in schools are adjusted. See Analysis section for details.

Fig. 1a shows mean total numbers of contacts by respondent age. Numbers of casual contacts per day were higher than numbers of close contacts per day in all age groups. Total close contact numbers varied little by respondent age. Total casual contact numbers were lowest in the youngest and oldest age groups.

**Fig. 2:**
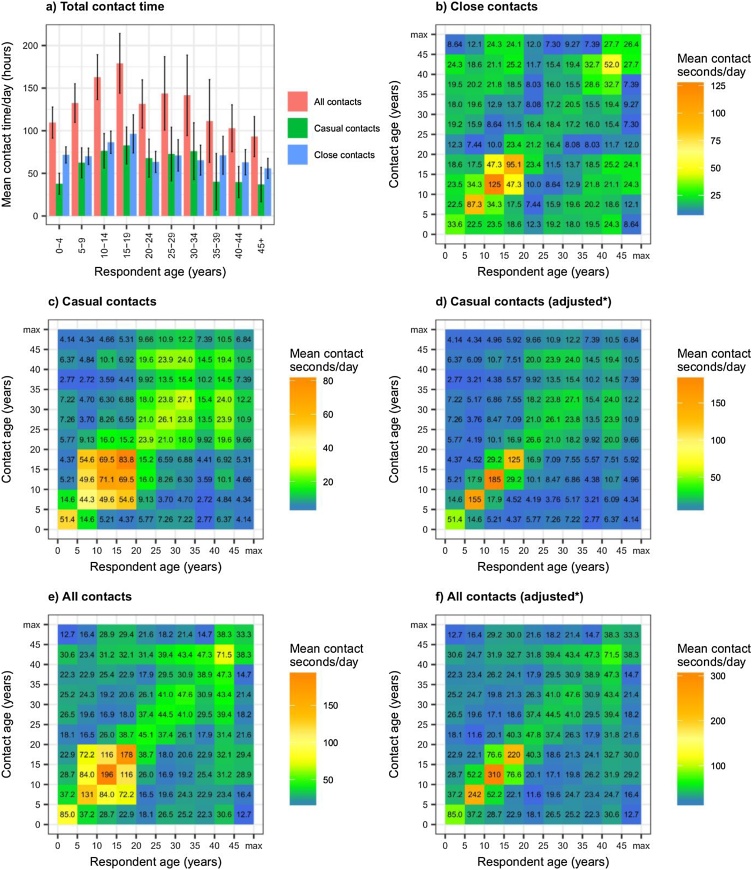
**Total contact time per day by respondent age group, and mean contact time between close, casual and all contacts per day by age group.** Contact times are the estimated mean number of seconds of contact per day between each individual in age group *a* with each individual in age group *b* (assuming a closed community). Graph a) shows total contact time by respondent age (summed over all of their contacts). Error bars show 95% confidence intervals, graph b) shows close contact times, graphs c) and d) show casual contact times, and graphs e) and e) show contact time in all contacts. *Age mixing patterns in schools are adjusted. See Analysis section for details.

Fig. 2a shows total contact time per respondent per day by respondent age group. Close and casual contact times were similar to each other within each respondent age group. Mean total contact times in adolescents and young adults were higher than in younger children and older adults.

**Fig. 3:**
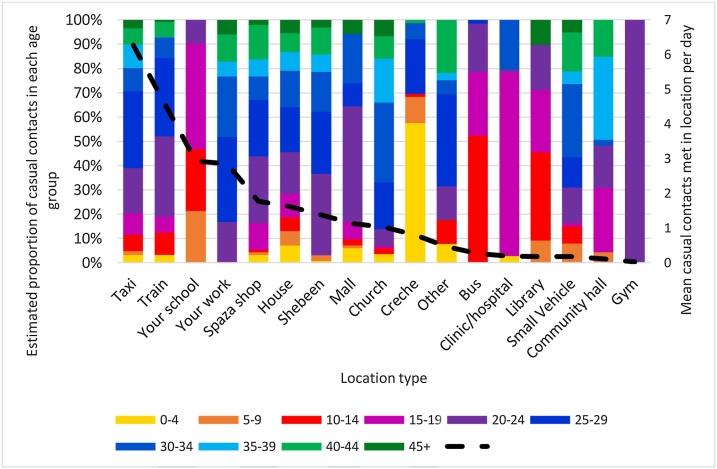
**Estimated proportion of casual contacts in each age group, and mean casual contacts met per day, by location type.** The bars show the estimated proportion of casual contacts present at a location type who are in each age group. The dotted line shows the mean number of casual contacts met by each respondents in locations of that type per day (including respondents who did not visit that location type).

Fig. 3 shows the mean number of casual contacts met by respondents in each location, and the estimated proportion of casual contacts who were in each age group, by type of location. The mean number of casual contacts was highest in minibus taxis and trains, followed by schools and workplaces. The estimated age distribution of casual contacts varied by location type. In many location types (e.g. taxis, trains, and shops), people in all age groups were present among casual contacts. In other location types (e.g. workplaces, schools, and crèches) only certain age groups were estimated to be present among casual contacts. Figure S2 shows the mean number of close and casual contacts present during a visit to a location, by location type.

### 3.2 Age-mixing

Fig. 1b–f shows age-mixing patterns among close, casual, and all contacts, calculated based on numbers of contacts. In this population, contacts were highly age-assortative among close, casual (adjusted in schools), and all contacts. The highest close, casual, and all contact rates were found between 5–9 year olds, 10–14 year olds, and 15–20 year olds within their own age groups.

Fig. 2b–f shows age-mixing patterns among close, casual, and all contacts, calculated based on contact time. As for contact numbers, contact patterns based on contact time were highly age assortative for close, casual, and all contacts, and the highest contact times were found between 5–9 year olds, 10–14 year olds, and 15–20 year olds within their own age groups. For close contacts, secondary diagonals can also be seen, reflecting high contact times between children and adolescents, and adults around 20–30 years older. A similar pattern has been found among close contacts in previous studies (Mossong et al., 2008). These secondary diagonals can not be seen in casual contacts, and have a lower relative magnitude in all contacts than they do in close contacts only.

For both contact numbers and contact time, adjusting estimates to account for highly assortative patterns of age-mixing in schools increased contact between children and adolescents in the same age group, and reduced contact between children and adolescents in different age groups (Figs. 1d, f, 2 d and f).

### 3.3 Sensitivity analyses

Age-mixing patterns between casual contacts in the three sensitivity analyses are shown in Figures S3 (contact numbers) and S4 (contact time). For all of the sensitivity analyses, there is little difference between the results of the main analysis and the results of the sensitivity analysis in age-mixing patterns in casual contact numbers or casual contact time.

## 4 Discussion

In this paper, we show that age-mixing patterns between casual contacts can be estimated using data that can be easily collected as part of social contact questionnaires. In this population, patterns of age-mixing based on contact numbers were similar between close, casual, and all contacts, however there is more variation by age group in total numbers of casual and all contacts than in total numbers of close contacts. Patterns of age-mixing based on contact time are more strongly assortative among casual and all contacts than among close contacts, with close contacts showing secondary diagonals between children/adolescents and adults around 20–30 years older.

Our findings demonstrate that estimated patterns of contact numbers and age-mixing patterns may differ between close and all contacts. While the differences are not large in this population, using mixing patterns from close contacts may nevertheless result in inaccurate model predictions. In other populations (e.g. settings with large numbers of contacts on public transport), differences in age-mixing patterns between close and all contacts may be larger, and the implications of using incorrect age-mixing patterns may be greater.

We used data from a social contact survey to estimate age-mixing patterns among close, casual, and all contacts. The same methods can also be used to estimate age-mixing patterns using data from time-use surveys, however among all contacts only. An attempt has also been made to estimate age-mixing patterns among close contacts using time-use data (Zagheni et al., 2008). Due to the assumptions behind the method however (that people have exactly one close contact at all times when they are in a location with other people, and that the age distribution of an individual’s close contacts is the same as the age distribution of all people present at that location type), age-mixing patterns among close contacts should be estimated directly when suitable data from contact surveys are available.

De Cao et al (De Cao et al., 2014) used data from both a contact survey and a time-use survey, producing a formula for combining age-mixing patterns from the two types of source, with a parameter, q_2_, controlling the relative importance that is given to each source. With high values of q_2_, their estimated age-mixing patterns are very similar to the patterns from the contact survey. With lower values of q_2_, their estimated age-mixing patterns are more similar to those from the time-use survey. They interpret their results as describing age-mixing patterns among *close* contacts only, with all close contacts being suitable for transmission in the former case, and only longer duration contacts in the latter case. An alternative, and perhaps more realistic, interpretation would be that the latter case describes age-mixing patterns suitable for droplet and close contact infections, and the former patterns suitable for airborne infections.

There are a number of assumptions that underlie our estimates of age-mixing between casual contacts. Firstly, the method assumes that patterns of age-mixing among casual contacts at a particular location type are proportional to the amount of time spent in that location type by people in each age group. This assumption will not be valid if individuals of different ages tend to visit different venues of a location type (e.g. young and older adults tend to frequent different bars), or if they tend to visit locations on different days of the week or at different times of day (e.g. working age adults tend use public transport in the early morning and evening, and older adults tend to use it outside rush hours). We tested the latter in a sensitivity analysis, by allowing the age distribution of casual contacts to vary by time of day (morning, afternoon, and evening), in addition to by location type. In this setting, estimating casual contact age-mixing within location types by time of day slightly increases the assortativeness of age mixing in 0–5 year olds and adults aged 40+ years, but appears to have little effect on overall patterns of age-mixing.

Due to strong patterns of assortative age-mixing in schools (due to age-stratified schools, and age-stratified classes within schools) we can be confident that the assumption that people of different ages do not visit different venues within a particular location type (e.g. younger and older adults frequenting different bars) is not valid for schools. We therefore assumed, for schools only, that patterns of casual age-mixing in schools mirrored patterns of close age-mixing in schools. Additional data collection is needed to measure the effects of the assumption on estimates for other location types in this study population. Where it seems likely *a priori* that the assumption is not valid for a particular location type, then estimates may be improved by subdividing the location type in the data collection tool (for instance by splitting ‘drinking place’ into ‘bar’ and ‘nightclub’).

An additional assumption that underlies this method is that the study community is closed, and that locations visited by study participants are only visited by other members of the study community. In practice, it does not matter if this condition is not met, provided that 1) the age distribution of people visiting locations of each type is the same for people from the study community as for people from outside the study community and/or 2) the majority of indoor contact that study participants have is with other members of the study community. In this study, 2) is unlikely to be true, as 58% of reported indoor casual contact time occurred in locations outside the study community. Further data collection is required to determine whether 1) is likely to be true for the study community.

Care needs to be taken that the sample size used is sufficient when using this method to estimate age-mixing patterns among casual contacts. Where only a small number of visits to a particular location type are reported, then it will not be possible to accurately estimate the age distribution of visitors to that location type. To illustrate, at its most extreme, with only one participant reporting visiting each location type, then the estimated age-mixing patterns among casual contacts will be 100% assortative. We demonstrated in a sensitivity analysis that excluding location types that fewer than 20 people reported visiting had little effect on our estimates of age-mixing in casual contacts, suggesting that our sample size was sufficient to obtain reasonable estimates of age-mixing. With a smaller sample size, or in a study population where a high proportion of casual contacts occur in infrequently visited locations, then this method may overestimate the assortativeness of age-mixing in casual contacts.

Finally, the questionnaire used in this study did not collect data on casual contacts made in locations where no close contacts occurred, which may have affected estimates of age-mixing. Future social contact questionnaires should ensure that indoor locations where only casual contacts are found are not neglected. In addition, further details on contact time occurring in workplaces should be collected, to enable more accurate estimates of the age distribution of casual contacts in workplaces that are not classed as workplaces by the majority of respondents (e.g. shops, which will be classed as ‘workplaces’ by shop workers and ‘shops’ by customers).

This method provides a valuable way of improving estimates of age-mixing for airborne infections such as *Mtb*. It requires minimal extra data collection beyond that typically collected in social contact studies. We have conducted a number of sensitivity analyses in this paper that demonstrate that some of the assumptions made do not bias the results, however other assumptions could not be tested without additional data collection. Due to the assumptions made by the method, it is important to think critically about the limitations when applying the method to other populations, and conduct sensitivity analyses where possible. In many cases however, the assumptions listed here may be more reasonable than the assumption implicitly made when estimating age-mixing patterns for airborne infection transmission from close contact data: that patterns of age-mixing among casual contacts are the same as patterns of age-mixing among close contacts. Modelling both close and casual contacts, making use of the more detailed data that can be collected on close contacts, may also be beneficial in some circumstances (McCreesh and White, 2018).

We would like to end this paper with the strong recommendation that researchers planning social contact studies include questions on the number of casual contacts present in indoor locations. While these data may not be necessary for researchers working on infections that are transmitted primarily or entirely through direct contact or droplet infections, they will assist greatly in the mathematical modelling of tuberculosis and other airborne infections.

## Contributions

NM designed and conducted the analysis, and wrote the paper. CM, KM and RW designed and conducted the original data collection. All authors revised the manuscript and approved the final version.

## Declaration of interests

None

## Funding source

This work was supported by the UK Medical Research Council (MRC) and the UK Department for International Development (DFID) under the MRC/DFID Concordat agreement that is also part of the EDCTP2 programme supported by the European Union MR/P002404/1. RGW is additionally funded by the Bill and Melinda Gates Foundation (TB Modelling and Analysis Consortium: OPP1084276/OPP1135288, SA Modelling for Policy: OPP1110334, CORTIS: OPP1137034, Vaccines: OPP1160830) and UNITAID (4214-LSHTM-Sept15; PO 8477-0-600). NM is supported by an MRC Skills Development Fellowship (MR/N014693/1). CM, KM, and RW are funded by the Strategic Health Innovation Partnerships (SHIP) Unit of the South African Medical Research Council, as a sub-grant from the Bill and Melinda Gates Foundation fund number OPP1116641.

The funders had no role in study design; in the collection, analysis and interpretation of data; in the writing of the report; and in the decision to submit the article for publication
