# Supplementary material for: Estimating age-mixing patterns relevant for the transmission of airborne infections
Source: Epidemics. 2019 Sep;28:100339. doi: 10.1016/j.epidem.2019.03.005 (PMC6731521; doi:10.1016/j.epidem.2019.03.005)
Supplement: Supplementary file 1 [file mmc1.pdf]

Figure S1. Diary used by participants to record all contacts that they met in a 24-hour period. The name of the study community has been blacked out

Participant ID □□□□□ Assigned Date □□/□□/□□□□

Place 1	□ In [redacted]<br>□ Out of [redacted]	□ Indoors<br>□ Outdoors	□ Your household<br>□ Other household on your plot<br>□ Outside on your plot<br>□ Other household off your plot<br>□ Street(s)<br>□ Taxi<br>□ Bus<br>□ Train<br>□ Spaza shop<br>□ Community hall<br>□ Soccer field<br>□ Your work: ________________<br>□ Your school: ________________<br>□ Crèche: ________________<br>□ Church: ________________<br>□ Clinic/hospital: ________________<br>□ Library: ________________<br>□ Gym: ________________<br>□ Shebeen: ________________<br>□ Other: ________________	Time spent:<br>□ 0 - 15min<br>□ 15min – 1hr<br>□ 1 – 2hrs<br>□ 2 – 4hrs<br>□ 4 - 8hrs<br>□ 8 – 12hrs<br>□ More than 12hrs	Time of day:<br>□ Morning<br>□ Afternoon<br>□ Evening	People touched or talked to: (see table below)

People touched or talked to:

Age	Gender	Touched	First time today
□□	□M □F	□Y □N	□Y □N
□□	□M □F	□Y □N	□Y □N
□□	□M □F	□Y □N	□Y □N
□□	□M □F	□Y □N	□Y □N
□□	□M □F	□Y □N	□Y □N
□□	□M □F	□Y □N	□Y □N
□□	□M □F	□Y □N	□Y □N
□□	□M □F	□Y □N	□Y □N
□□	□M □F	□Y □N	□Y □N
□□	□M □F	□Y □N	□Y □N
□□	□M □F	□Y □N	□Y □N
□□	□M □F	□Y □N	□Y □N
□□	□M □F	□Y □N	□Y □N
□□	□M □F	□Y □N	□Y □N
□□	□M □F	□Y □N	□Y □N
□□	□M □F	□Y □N	□Y □N
□□	□M □F	□Y □N	□Y □N
□□	□M □F	□Y □N	□Y □N
□□	□M □F	□Y □N	□Y □N
□□	□M □F	□Y □N	□Y □N
□□	□M □F	□Y □N	□Y □N
□□	□M □F	□Y □N	□Y □N
□□	□M □F	□Y □N	□Y □N
□□	□M □F	□Y □N	□Y □N
□□	□M □F	□Y □N	□Y □N

<u>If indoors</u>
People not touched or talked to:
Number: ___________________

Figure S2. Estimated mean close and casual contacts present during a visit to a location, by location type.

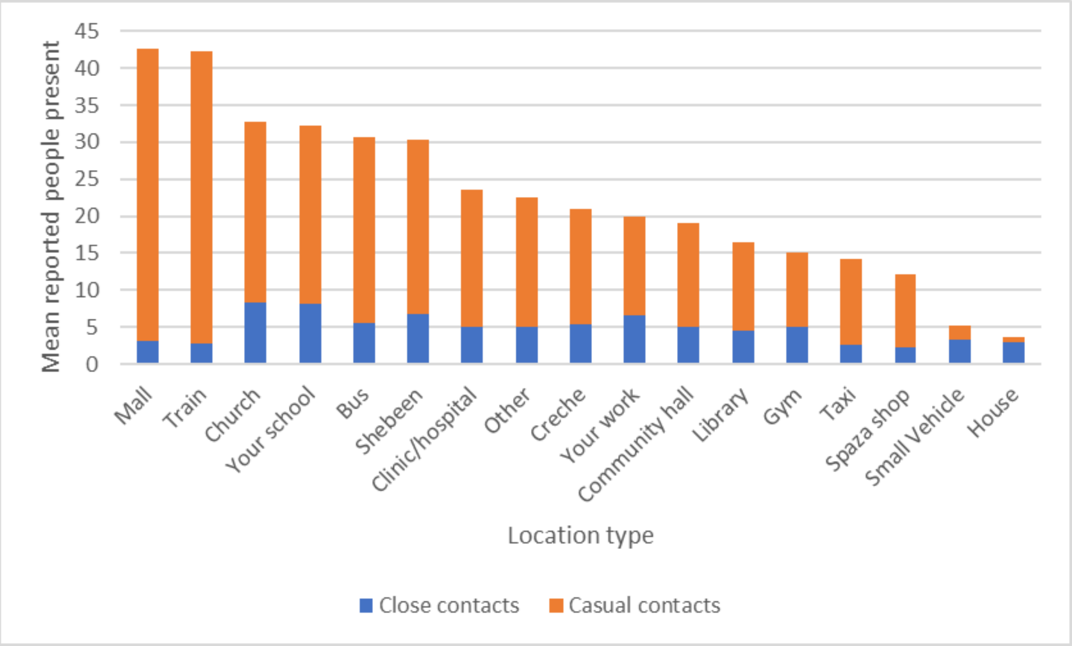

Figure S3. Estimated casual contact rates between different age groups in the main analysis and three sensitivity analyses. Rates are the estimated rate of contact per day between each individual in age group *a* with each individual in age group *b* (assuming a closed community). Graphs a) and b) show results from the main analysis. Graphs c) and d) show results when location types that fewer than 20 people reported visiting were excluded from the analysis. Graphs e) and f) show results when the proportions of people present in each type of location who were in each age group were estimated *by time of day* (morning, afternoon, or evening). Graphs g) and h) show results when the age distribution of casual contacts in workplaces was estimated from the age distribution of close contacts that respondents reported contacting in workplaces. *Age mixing patterns in schools are adjusted. See Analysis section for details.

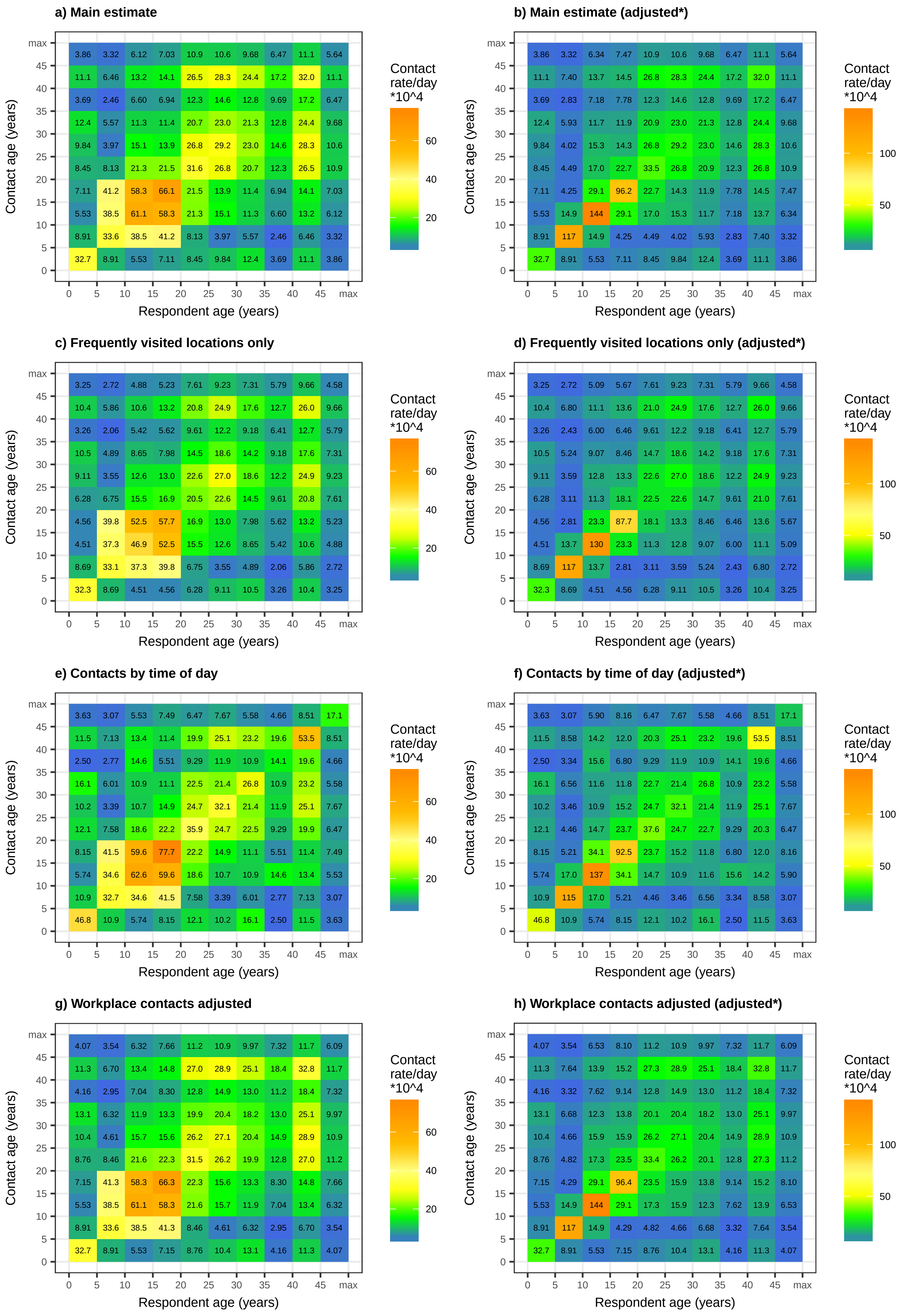
a) Main estimate
b) Main estimate (adjusted*)
c) Frequently visited locations only
d) Frequently visited locations only (adjusted*)
e) Contacts by time of day
f) Contacts by time of day (adjusted*)
g) Workplace contacts adjusted
h) Workplace contacts adjusted (adjusted*)
Contact age (years)
Respondent age (years)
Contact rate/day *10^4

Figure S4. Estimated mean contact time between different age groups in the main analysis and three sensitivity analyses. Contact times are the estimated mean number of seconds of contact per day between each individual in age group *a* with each individual in age group *b* (assuming a closed community). Graphs a) and b) show results from the main analysis. Graphs c) and d) show results when location types that fewer than 20 people reported visiting were excluded from the analysis. Graphs e) and f) show results when the proportions of people present in each type of location who were in each age group were estimated *by time of day* (morning, afternoon, or evening). Graphs g) and h) show results when the age distribution of casual contacts in workplaces was estimated from the age distribution of close contacts that respondents reported contacting in workplaces. *Age mixing patterns in schools are adjusted. See Analysis section for details.

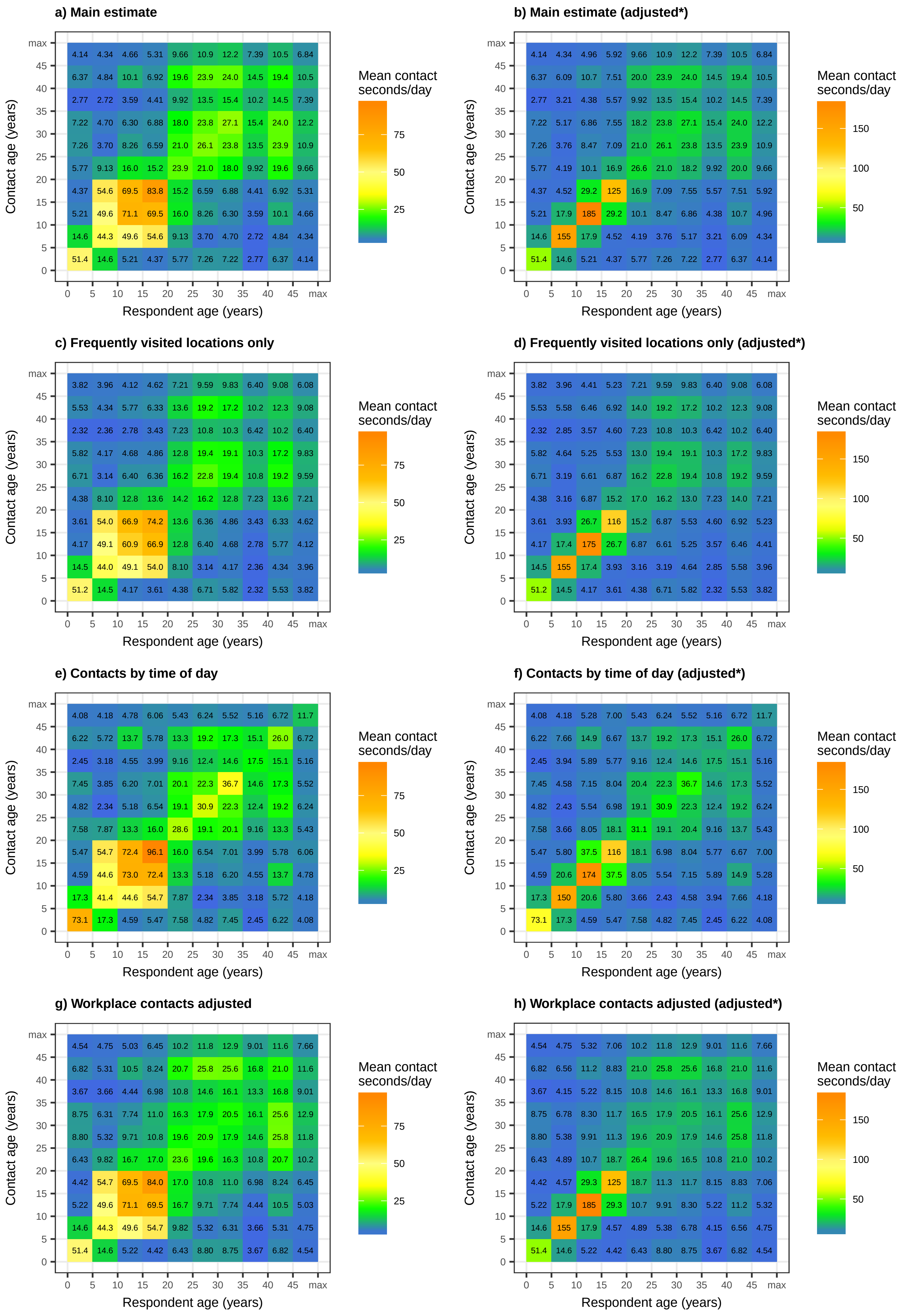
a) Main estimate
b) Main estimate (adjusted*)
c) Frequently visited locations only
d) Frequently visited locations only (adjusted*)
e) Contacts by time of day
f) Contacts by time of day (adjusted*)
g) Workplace contacts adjusted
h) Workplace contacts adjusted (adjusted*)
Contact age (years)
Respondent age (years)
Mean contact seconds/day
0
5
10
15
20
25
30
35
40
45
max
25
50
75
100
150
